# Delays in Coccidioidomycosis Diagnosis and Associated Healthcare Utilization, Tucson, Arizona, USA

**DOI:** 10.3201/eid2509.190023

**Published:** 2019-09

**Authors:** Fariba M. Donovan, Patrick Wightman, Yue Zong, Luke Gabe, Aneela Majeed, Tiffany Ynosencio, Edward J. Bedrick, John N. Galgiani

**Affiliations:** University of Arizona, Tucson, Arizona, USA (F.M. Donovan, P. Wightman, L. Gabe, A. Majeed, T. Ynosencio, E.J. Bedrick, J.N. Galgiani);; Valley Fever Center for Excellence, Tucson (F.M. Donovan, Y. Zong, J.N. Galgiani)

**Keywords:** Valley fever, coccidioidomycosis, delay in diagnosis, healthcare cost, antimicrobial drugs, fungi, Tucson, Arizona

## Abstract

Tucson, Arizona, USA, is a highly coccidioidomycosis-endemic area. We conducted a retrospective review of 815 patients in Tucson over 2.7 years. Of 276 patients with coccidioidomycosis, 246 had a delay in diagnosis; median delay was 23 days. Diagnosis delay was associated with coccidioidomycosis-related costs totaling $589,053 and included extensive antibacterial drug use.

Coccidioidomycosis, also known as Valley fever, is a fungal infection endemic to the southwestern United States and Mexico (*1*); it typically manifests as a respiratory syndrome (*2*). Without specific laboratory confirmation, coccidioidomycosis cannot be distinguished from community-acquired pneumonia (*3*,*4*). However, the necessary tests are conducted for <13% of patients with community-acquired pneumonia in urban Arizona ambulatory clinics (*5*) and 2.8% of patients in emergency departments (*6*). Until coccidioidomycosis is correctly diagnosed, patients are likely to receive unnecessary antibacterial drugs, laboratory tests, imaging, and invasive procedures, all of which could contribute to unnecessary costs and additional adverse health consequences. The extent to which there is a delay in coccidioidomycosis diagnosis and how much healthcare utilization occurs with that delay is uncertain.

We report a retrospective study within metropolitan Tucson, Arizona, USA, an area in which coccidioidomycosis is highly endemic, to determine the delay for patients from the date when they first sought care to the date they received a laboratory-confirmed coccidioidomycosis diagnosis. We also analyzed healthcare costs, efforts, and antibacterial drug use within that period.

## The Study

We conducted a retrospective review of medical charts from January 1, 2015, through September 18, 2017, for patients at Banner University Medical Center in Tucson (BUMC-T). During this time, BUMC-T used the Epic electronic medical record system (*7*). The University of Arizona Health Sciences Clinical Data Warehouse (CDW; https://cb2.uahs.arizona.edu/clinical-data-warehouse) provided the information. Two physicians, an infectious disease fellow (A.M.) and pulmonology clinical fellows (L.G., T.Y.), independently reviewed charts with codes 114.*/B38.* from the International Classification of Diseases, 9th Revision (ICD-9) or 10th Revision (ICD-10). We divided records into 2 cohorts and, when combined, sampled the entire study period. Two different reviewers were assigned to each cohort. We excluded records if coccidioidomycosis was diagnosed before the study, if the ICD-9/10 coding was in error, if patient age was <18 years, or if diagnosis was not confirmed by laboratory testing (*2*). For the remaining records, we determined initial presentation date (i.e., the date signs and symptoms were first clinically noted). We categorized presenting syndromes by type: acute symptomatic pulmonary infection or immunologic response (rash, arthralgia, fatigue), fibrocavitary chronic pulmonary infection, asymptomatic pulmonary nodule, and extrapulmonary disseminated infection. The specimen collection date leading to laboratory confirmation was designated as the diagnosis date and the time between initial evaluation and diagnosis date as the diagnosis delay. Agreement between reviewers for delay was 98% and 96.5% in the 2 cohorts; the lead investigator (F.M.D.) resolved discrepancies.

Within the range for diagnostic delay, we queried the CDW for Healthcare Common Procedure Coding System/Current Procedural Terminology (HCPCS/CPT) codes to estimate total effort (i.e., all CPTs associated with each encounter). We used publicly available Medicare fee schedules assigning values to codes associated with charges. These included the Physician Fee Schedule, Clinical Laboratory Fee Schedule (Arizona-specific), Payment Allowance for Medicare Part B Drugs, and the Durable Medical Equipment, Prosthetics/Orthotics, and Supplies (DMEPOS) Fee Schedule. We used 2016 schedules to correspond with the last full year that Banner used Epic software and to impose a uniform price scheme. We manually reviewed each CPT code and tabulated costs related to coccidioidomycosis diagnosis; costs are presented in US dollars. The University of Arizona institutional review board approved this study.

**Figure 1:**
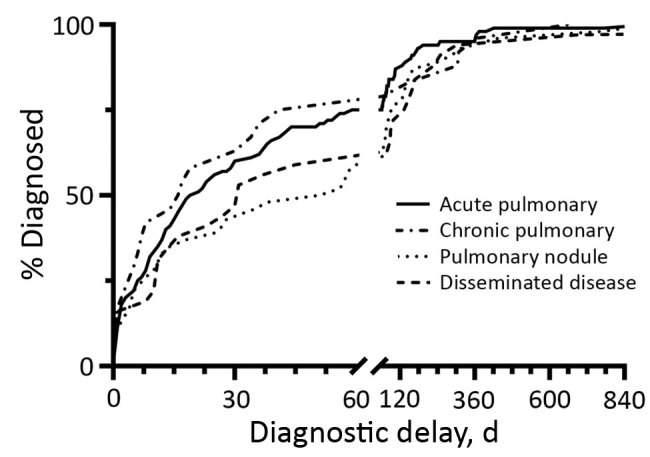
Cumulative distribution for time to diagnosis of coccidioidomycosis for 4 disease categories among patients in Tucson, Arizona, USA, January 1, 2015–September 18, 2017. Time points related to patients with very extended delay period (909 days in acute pulmonary disease, 928 days in pulmonary nodule, and 3,315 days in disseminated disease) are not shown in the graph. Timeline truncated for readability.

**Figure 2:**
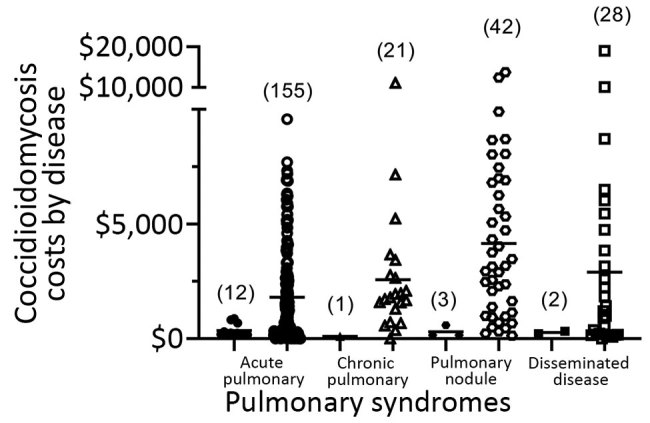
Coccidioidomycosis costs by disease category for 264 case-patients in Tucson, Arizona, USA, January 1, 2015–September 18, 2017. Costs are shown in 2 columns for each category: the left column for diagnosis at initial presentation and the right column for delayed diagnosis. Each symbol represents 1 case. Numbers in parentheses indicate the number of cases in each category. Cost axis truncated for readability.

We summarized categorical variables using counts and percentages and summarized time to events and costs using medians and interquartile ranges (Figures 1, 2). We showed total costs by disease category. We compared the distributions of time to diagnosis and costs across coccidioidomycosis presentation categories using nonparametric Kruskal-Wallis tests. We based pairwise comparisons of groups on nonparametric Wilcoxon-Mann-Whitney tests and CIs for differences in medians. We used a χ^2^ test to compare percentages between groups. We conducted statistical analyses in R version 3.1 (https://www.r-statistics.com/2014/11/r-3-1) and defined statistical significance as p<0.05.

We reviewed a total of 815 electronic charts in 2 cohorts and excluded 539 (66%) of them (Appendix 1 Table 1). Exclusions were for prior coccidioidomycosis (57%), mistaken coding (11%), age <18 years (10%), and unconfirmed diagnosis (22%). Of 276 remaining cases, 126 (46%) were in female patients; average patient age was 55.5 years (SD + 18.4), and median age was 53.3 years. Disease category distribution was acute symptomatic pulmonary (63%), chronic pulmonary (8%), asymptomatic pulmonary nodule (17%), and disseminated infection (12%). We found no significant difference (p = 0.38) between excluded and included cases in the 2 cohorts. 

We compiled cumulative distribution functions for time to diagnosis (Figure 1). Differences in delay among disease categories were not statistically significant (p = 0.14). Median delay was 23 days (95% CI 17–34), and 43% of patients (95% CI 38–50) had a delay >1 month.

Of 276 patients, 30 (11%) received a coccidioidomycosis diagnosis at presentation (zero delay) and 12 of the 30 during hospital admission. Because hospitalization of these 12 either was not required for diagnosis or was required for disease complication management, we excluded coccidioidomycosis-associated costs from the analysis. Of the remaining 264 patients, 246 had delayed diagnosis of >1 day, with coccidioidomycosis-associated costs of $589,053 (82%) of the $718,401 total. Coccidioidomycosis-related costs for patients with diagnostic delay were significantly greater (p = 0.0004) than for the 18 patients without delay (Figure 2; Appendix 1 Table 2).

Not all CPT codes are associated with charges or reflected in patients’ bills, so we assessed total effort by analyzing the frequency of CPT codes and determining whether codes were related to coccidioidomycosis infection (Appendix 2). Coccidioidomycosis-related charges for CPT code–related effort was higher in patients with delays in diagnosis compared with charges for patients without delays (Appendix 1 Figure 1).

Outpatient and inpatient prescriptions for a total of 1,103 antibacterial medication orders submitted before coccidioidomycosis diagnosis are an approximate measure of antibacterial use. Vancomycin and daptomycin comprised 22% of antimicrobial drugs ordered (Appendix 1 Figure 2).

## Conclusions

Our findings demonstrate significant delays in accurate coccidioidomycosis diagnosis and substantial costs associated with these delays. Median delays for different disease categories ranged from 17 to 54 days; 43% of patients with coccidioidomycosis had a diagnosis delay >1 month. The 23-day median delay we presented (interquartile range 7–74 days) corresponds with a recently reported 23-day delay in Arizona (*8*) and in a 2010 study (*9*). In our cohort, these delays were associated with $589,053 in coccidioidomycosis-related costs, as determined from Medicare fee schedules. Although median coccidioidomycosis-related costs were lowest for acute coccidioidal pneumonia, the total was greatest for this group because it accounted for >63% of patients. If our findings were extrapolated across institutions in coccidioidomycosis-endemic regions, diagnostic delays and excess healthcare utilization would probably represent millions of dollars. 

Overall healthcare effort as indicated by CPT code frequencies, irrespective of whether or not they resulted in billed charges, showed similar delay-associated excesses. Of additional concern is unnecessary use of antibacterial drugs, such as broad-spectrum medications like vancomycin, in coccidioidomycosis patients before an accurate diagnosis (Appendix 1 Figure 2). If coccidioidomycosis diagnostic delays were shortened, unnecessary antibacterial treatments could be reduced greatly.

This study extends earlier reports of the economic burden associated with coccidioidomycosis (*9*). It is certainly an underestimate of costs, because we did not include in our cohort an unknown number of patients with coccidioidomycosis who were misdiagnosed. Our results suggest earlier diagnosis will lower costs and provide secondary benefits including patient reassurance, decreased antibacterial drug use, and improved antibiotic stewardship. This study reinforces the ongoing challenge to increase coccidioidomycosis awareness for healthcare providers and the urgent need to improve the ease, rapidity, and reliability of coccidioidomycosis testing.

Appendix 1 Additional information about delays in coccidioidomycosis diagnosis, Tucson, Arizona, USA.

Appendix 2 CPT codes and their assignment as coccidioidomycosis related (Yes) or unrelated (No).
