# Supplementary material for: Delays in Coccidioidomycosis Diagnosis and Associated Healthcare Utilization, Tucson, Arizona, USA
Source: Emerg Infect Dis. 2019 Sep;25(9):1745–7. doi: 10.3201/eid2509.190023 (PMC6711243; doi:10.3201/eid2509.190023)
Supplement: Appendix 1 — Additional information about delays in coccidioidomycosis diagnosis, Tucson, Arizona, USA. [file 19-0023-Techapp-s1.pdf]

Article DOI: https://doi.org/10.3201/eid2509.190023

Delays in Coccidioidomycosis Diagnosis and Associated Healthcare Utilization, Tucson, Arizona, USA

Appendix

Appendix Table 1. Patients in each of 2 cohorts of coccidioidomycosis study excluded for specific reasons and included by category of disease, Tucson, Arizona, USA

Characteristic	Cohort 1, no. (%)	Cohort 2, no. (%)	Total (%)
Excluded, by reason			
History of coccidioidomycosis	138 (57)	169 (57)	307 (57)
Mistaken coding	35 (14)	24 (8)	59 (11)
Age <18 y	27 (11)	29 (10)	56 (10)
Unconfirmed diagnosis	44 (18)	73 (25)	117 (22)
Total	244 (68)	295 (65)	539 (66)
Included, by disease			
Acute symptomatic pulmonary	72 (62)	102 (64)	174 (63)
Chronic pulmonary	16 (14)	8 (5)	24 (8)
Pulmonary nodule	18 (15)	28 (17)	46 (17)
Disseminated disease	10 (9)	22 (14)	32 (12)
Total	116 (32)	160 (35)	276 (34)
Total charts reviewed	360	455	815

Appendix Table 2. Diagnostic delays and related costs for patients with coccidioidomycosis, by category of disease, Tucson, Arizona, USA

Characteristic	Acute symptomatic pulmonary	Chronic pulmonary	Pulmonary nodule	Disseminated	Total
Total no. patients	N = 174	N = 24	N = 46	N = 32	N = 276
Median delay, d	19	17	54	31	23
Interquartile range of delay, d	6–61	4–47	9–93	10–137	7–74
No. patients with no delay in diagnosis (total)*	12 (18)	1 (4)	3 (4)	2 (4)	18(30)
Median coccidioidomycosis-related cost (total cost†)	$237 ($237)	$96 ($96)	$152 ($471)	$271 ($276)	$226 ($237)
Total coccidioidomycosis-related cost (total cost†)	$4,264 ($4,303)	$96 ($96)	$892 ($1,306)	$542 ($553)	$5,794 ($6,258)
No. patients with diagnosis delayed ≥1 d	156	20	42	28	246
Median delay, d	24	19	57	41	30
Interquartile delay, d	10 - 67	7 - 64	12–118	15 –150	10–82
Median coccidioidomycosis-related cost (total cost†)	$918 ($1,107)	$1,796 ($1,877)	$3,157 ($4,266)	$1,094 ($1,608)	$1,419 ($1,633)
Total coccidioidomycosis-related cost (total cost†)	$279,740 ($335,434)	$53,985 ($56,844)	$174,269 ($222,066)	$81,059 ($97,799)	$589,053 ($712,143)

*This cost analysis includes 18 of the 30 patients because hospital costs associated with 12 patients were not required for diagnosis. For each category of disease, the number of patients with immediate ambulatory care diagnosis and the total number of patients in that category are shown.
†Total hospital costs include coccidioidomycosis-related and non–coccidioidomycosis-related expenses.

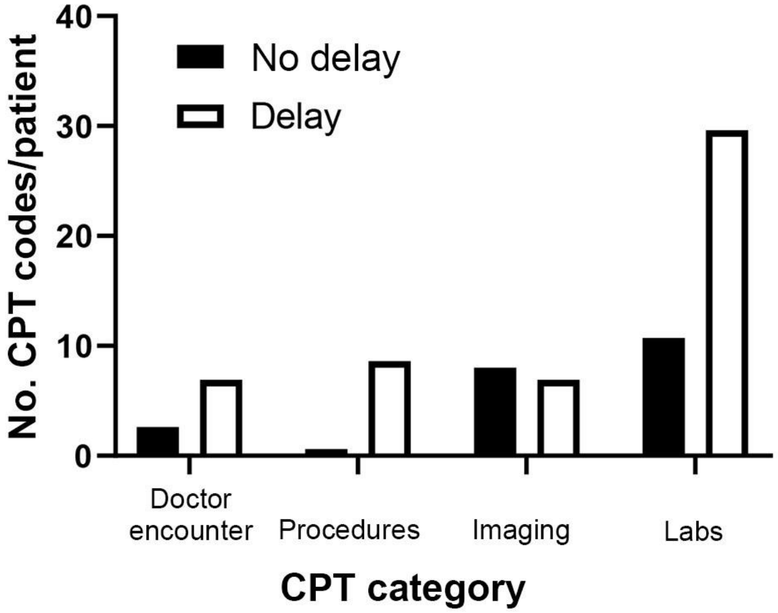

Appendix Figure 1. Coccidioidomycosis-related CPT codes by category for patients with (white) and without (black) delays in diagnosis.

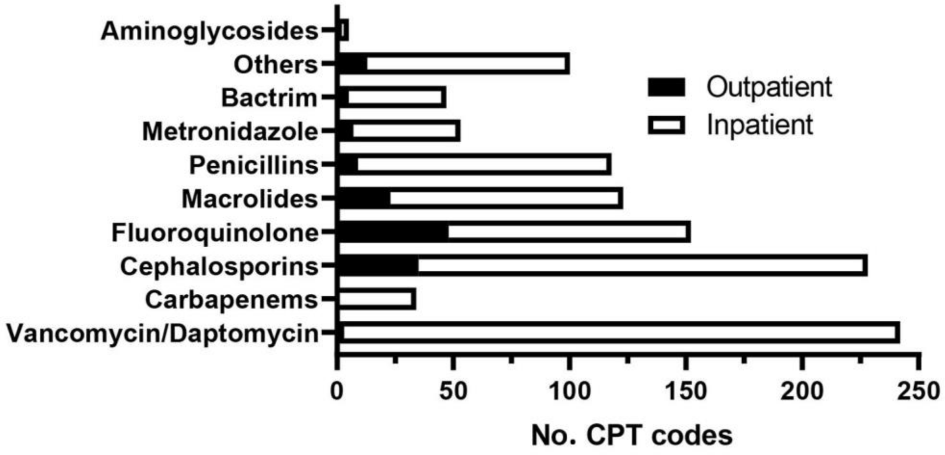

Appendix Figure 2. Use of antibacterial drugs before diagnosis for inpatient (white) and outpatient (black) care of patients with coccidioidomycosis infection, Tucson, Arizona, USA.
